# Causation of Acute Flaccid Paralysis by Myelitis and Myositis in Enterovirus-D68 Infected Mice Deficient in Interferon αβ/γ Receptor Deficient Mice

**DOI:** 10.3390/v10010033

**Published:** 2018-01-12

**Authors:** John D. Morrey, Hong Wang, Brett L. Hurst, Katherine Zukor, Venkatraman Siddharthan, Arnaud J. Van Wettere, Donal G. Sinex, E. Bart Tarbet

**Affiliations:** 1 Institute for Antiviral Research, Department of Animal, Dairy, and Veterinary Sciences, 5600 Old Main Hill, Utah State University, Logan, UT 84322, USA; wang.hong@aggiemail.usu.edu (H.W.); brett.hurst@usu.edu (B.L.H.); Katherine.zukor@usu.edu (K.Z.); venkat.siddharthan@usu.edu (V.S.); bart.tarbet@usu.edu (E.B.T.); 2 Utah Veterinary Diagnostics Laboratory, Department of Animal, Dairy, and Veterinary Sciences, 950 East 1400 North, Utah State University, Logan, UT 84341, USA; arnaud.vanwettere@aggiemail.usu.edu; 3 Department of Communication Disorders and Deaf Education, 2800 Old Main Hill, Utah State University, Logan, UT 84322, USA; donal.sinex@aggiemail.usu.edu

**Keywords:** enterovirus, enterovirus-D68, paralysis, myelitis, myositis, electrophysiology, spinal cord, motor neurons, muscle atrophy

## Abstract

Enterovirus D68 (EV-D68) caused a large outbreak in the summer and fall of 2014 in the United States. It causes serious respiratory disease, but causation of associated paralysis is controversial, because the virus is not routinely identified in cerebrospinal fluid. To establish clinical correlates with human disease, we evaluated EV-D68 infection in non-lethal paralysis mouse models. Ten-day-old mice lacking interferon responses were injected intraperitoneally with the virus. Paralysis developed in hindlimbs. After six weeks of paralysis, the motor neurons were depleted due to viral infection. Hindlimb muscles were also infected and degenerating. Even at the earliest stage of paralysis, muscles were still infected and were degenerating, in addition to presence of virus in the spinal cord. To model natural respiratory infection, five-day-old mice were infected intranasally with EV-D68. Two of the four infected mice developed forelimb paralysis. The affected limbs had muscle disease, but no spinal cord infection was detected. The unique contributions of this study are that EV-D68 causes paralysis in mice, and that causation by muscle disease, with or without spinal cord disease, may help to resolve the controversy that the virus can cause paralysis, even if it cannot be identified in cerebrospinal fluid.

## 1. Introduction

Enterovirus D68 (EV-D68) is recognized as a significant emerging picornavirus, especially after a large and widespread outbreak primarily among children in the summer and fall of 2014 in the United States [1]. EV-D68 was originally classified as respiratory rhinovirus 87 due to its heat- and acid-lability, and optimal growth temperature of 33 °C. It was reclassified, however, as an enterovirus due to sequence molecular analysis [2]. EV-D68 preferentially replicates in the upper respiratory tract and spreads via respiratory secretions, unlike other heat- and acid-stable enteroviruses that preferentially replicate in the gastrointestinal tract and spread by the fecal-oral route. Beginning in 2008, small clusters of upper respiratory cases began to emerge. Cases continued to increase worldwide, leading to the 2014 outbreak.

Increases of unexpected incidences of acute flaccid myelitis (AFM) [3,4,5] coincided with outbreaks of EV-D68-induced severe respiratory disease [6,7,8]. This prompted concerns that EV-D68 is neurotropic and causes AFM, perhaps like poliovirus and enterovirus 71. However, the rhinovirus-like characteristics and the inability to isolate EV-D68 from the cerebrospinal fluid (CSF) and blood of patients with AFM are caveats to this hypothesis. Nevertheless, epidemiological and genetic evidences still support the causation of AFM by EV-D68.

In 2012 to 2015, California reported increased incidences of acute flaccid limb weakness with lesions in the spinal cord gray matter on magnetic resonance imaging (MRI) or as identified by electrophysiology [9]. In August 2014, during EV-D68 respiratory outbreak, a cluster of children with a similar neurological illness was identified in Colorado [5]. In a broader report [10], 120 children from 34 states met the criteria for AFM between August to December of 2014. Additional reports continued to describe the association of AFM with the 2014 EV-D68 outbreak [11,12].

Despite the lack of identification of EV-D68 in the CSF of AFM cases, EV-D68 is still considered by some to be the best candidate for causing the increased incidences of AFM. Evidence involves the detection of EV-D68 in respiratory secretions of 64% of patients with AFM at the height of the 2014 outbreak. EV-D68 was detected for the first time in the blood of a child with AFM using metagenomic sequencing and RT-PCR [13]. In the same study, phylogenetic analysis revealed that all EV-D68 sequences that were associated with AFM were of clade B1, where 5 of 6 coding polymorphisms were present in neuropathic poliovirus and/or enterovirus D70. EV-D68 was considered to be the most likely cause of associated AFM. No other infectious agents of clinical significance were identified in CSF of any 2012–2105 AFM patients using extensive next-generation sequencing and microbiological investigations, with one exception, where EV-D68 was identified in the CSF from a bloody lumbar puncture of one patient [13].

In the current study, using electrophysiological and histological approaches, we demonstrate that EV-D68 causes motor deficits in mice that are similar to human AFM. To develop a suitable neurological EV-D68 mouse model, we considered that humans are susceptible to some viruses, because these viruses can inhibit human interferon pathways [14]. However, mice may not be susceptible to these viruses, if the viruses cannot inhibit mouse-specific interferon pathways [15]. Indeed, the initial main reason for using AG129 mice is that they are susceptible to disease, but adult or adolescent wild-type C57BL/6 are not. Since many viruses, including enteroviruses [16,17], and more specifically, EV-D68 [18,19], can inhibit interferon responses to gain advantages in the human host, we fortuitously established that EV-D68 infection causes disease in AG129 mice lacking interferon type-I and -II receptors. Intraperitoneal (i.p.) injection of P2 neonatal Swiss Webster mice can cause paralysis [20], but AG129 mice are required to achieve paralysis in 5- and 10-day-old mice of this study analogous to human children infected with EV-D68 [9].

The objective of this study, therefore, was to identify neurological and muscle disease that might contribute to paralysis in AG129 mice challenged i.p. or intranasally with EV-D68 and to establish clinical correlates with human disease.

## 2. Materials and Methods

### 2.1. Ethics Statement

All of the experimental procedures were performed in compliance with animal protocols (#2125, 11 March 2014) approved by the Institutional Animal Care and Use Committee at Utah State University and in compliance with the “Guide for the Care and Use of Laboratory Animals” [21].

### 2.2. Animals

This work was done in the Association for Assessment and Accreditation of Laboratory Animal Care International-accredited, biosafety level 2 laboratory at Utah State University. Only male AG129 mice [22] were used and were bred in-house in sterilized cages and maintained in a 12/12 light cycle. Animals were euthanized with an overdose of pentobarbital.

### 2.3. Viruses

The viral strain used was EV-D68 US/MO/14-18949, clade B. It was passaged twice in rhabdomyosarcoma (RD) cells to generate a stock for injection. Ten-day-old mice were injected intraperitoneally (i.p.) with 10^6.8^ 50% cell culture infectious doses (CCID50) in a volume of 50 µL, only on the right side inguinal area. To prepare stock for intranasal installation of five-day-old mice, the virus was passaged 30 times by intranasal installation in four-week-old AG129 mice (manuscript in preparation). A volume of 90 µL with 1 × 10^6.5^ CCID50 was distributed between nares with every passage.

### 2.4. Motor Function Assays

Two different motor function assay were used, because two different collaborating laboratories measured motor function of mice.

### 2.5. Viral Paresis Scale (VPS)

VPS measurements were performed by a technician who was blinded to the identity of the mice. Mice were analyzed for signs of tail and hindlimb paresis/paralysis using a sensitive, open-field assay that was modified from the Basso Mouse Scale used to assess paralysis in spinal cord injured mice [23], and a test used to track paralysis in amyotrophic lateral sclerosis mouse models [24]. The specific VPS assay is described [25]. Each mouse was placed on a tabletop and allowed to roam freely for four minutes. Hindlimb function was scored on a seven-point scale from normal to total paralysis by researchers who were blind to the infection status of each group. Scoring was based on four main categories: tail position during walking, miss-step severity, weight bearing, and joint movement. Miss-step severity was scored only on assessable walking passes, which was defined as a pass in which the animal moved three body lengths at a consistent speed and without turning [23]. A score of 0 = normal; 1 = onset of symptoms; 2 = mild paresis; 3 = moderate paresis; 4 = severe paresis; 5 = paralysis; and 6 = complete paralysis. Separate scores were given for the left (contralateral) and right (ipsilateral) hindlimbs to assess if symptoms were bilateral or unilateral. Herein, we refer to the right side as the ipsilateral side, and the left side as the contralateral side.

### 2.6. Compound Muscle Action Potential (CMAP) and Nerve Conduction Velocity (NCV)

To measure the electrophysiological function of populations of axons and neuromuscular junctions, CMAP was performed. AG129 mice, challenged i.p. with virus 6 weeks earlier at 10 days of age, were anesthetized, were anesthetized with isoflurane (~1.5% isoflurane for 20-g mouse when shearing hair, injecting, and recording spontaneous activities, ~1% for maintenance, CMAP, and f-wave recordings). Since electrophysiology is sensitive to temperature variation, temperature of mice was maintained (37.0 ± 0.2 °C) with a heating pad with a feed-back rectal probe. To avoid electrical interference with some procedures, the heating pad was temporarily turned off during measurements. Both of the hindlimbs were sheared and hair removed with Nair^™^. Skin was cleaned and oils removed with 70% ethanol.

Measurements were performed by an investigator who knew the design of the experiments and could not be blinded to the identity of the mice, because all of the mice had at least one paralyzed hindlimb when compared to the sham controls. Accupunture needles (Tai Chi Brand, Lhass Medical, Inc., Accord, MA, USA) used as monopolar needle electrodes were inserted into the sciatic notch to stimulate the gastrocnemius muscle with 0.1-ms pulses of current using a stimulus isolator (WPI Isostim A320, World Precision Instruments, Inc., Sarasota, FL, USA). Ring electrodes were placed around the belling of the muscle and the tendons just below the muscle of the outstretched leg and connected to a differential amplifier (DAM 50, WPI) with a gain of 100× and filtered with a 300-1 K Hz band pass filter. Data were acquired at a 20 K/s sampling rate with Powerlab 4/25 and LabChart 8 software (ADInstruments, Sydney, Australia). Responses to 5 pulses at 1 Hz were averaged, and the current was increased incrementally until a maximum amplitude was reached. Maximal CMAP amplitudes were measured from peak to peak of the M-wave.

To measure the conductivity of axons, NCV was calculated by measuring the latency time of the peak CMAP using monopolar electrodes that were recorded from the foot plantar muscle after stimulation at the sciatic notch and tibial nerve posterior to the medial malleolus (ankle) [26]. Measurements from maximum amplitude peaks were used. The NCV was the distance between the two stimulation sites divided by the difference in latencies between the sciatic notch and tibial nerve stimulation sites. Values were reported as m/s.

### 2.7. F-Wave

To evaluate nerve conduction problems at or near the spinal cord, F-wave assay was performed. A stimulating electrode was kept in place at ankle, while the ring electrodes and leg restraints were removed. The toe was taped to the bench. Monopolar active recording electrode was inserted in the proximal side of the 4th interosseous muscle and the reference monopolar electrode was inserted into the distal side of the 4th interosseous muscle. The ground electrode was placed at the base of the tail, as described [27]. The frequency of stimulus was to 0.2–0.25 Hz and the number of stimuli was 3. As with CMAP, increase the stimulus (beginning at 0.3 mA) until a maximum response was obtained. The F-wave was identified from the various tracings, and the latency was recorded as the time of stimulation to the inflexion of response. The shortest latency was reported.

### 2.8. Spontaneous EMG Activity

To delineate types of peripheral nerve abnormalities, spontaneous EMG activities were determined. All the recording and stimulating needles were removed, and the isoflurane was elevated to 1.5%. Amplifier filter was changed to 10–10,000 Hz. The ground was inserted into the muscle at the base of the tail and clipped to a common ground. Complete anesthesia was ensured with ear/forepaw pinch test. After starting the recording, a disposable concentric needle electrode (#9013S0022 Dantec^™^ DCN, Natus Neurology, Middleton, WI, USA) was inserted into middle of the body of the gastrocnemius muscle to observe insertional activity. Duration of each recording was 10 min. Tracings were then examined for diagnostic spontaneous activities. All the tests were done on both hindlimbs.

### 2.9. Histological Analyses

Mice were perfused transcardially with phosphate buffered saline (PBS), followed by 4% paraformaldehyde. Tissues were removed and post-fixed in the same fixative overnight, rocking at 4 °C. Tissues were rinsed twice in PBS and the lumbosacral spinal cord, sciatic nerves, and gastrocnemius muscles were isolated. All the tissues were cryoprotected in 30% sucrose in PBS for 2–3 days at 4 °C, embedded in optimal cutting temperature (OCT) compound (Ted Pella, Redding, CA, USA), and frozen with a dry ice/isopentane bath. Five sets of adjacent sections were cut on a cryostat at 25 μm, mounted on slides, and stored at −20 °C until being ready for further processing. For immunohistofluorescence staining, sections were encircled with a hydrophobic barrier pen (ImmEdge, Vector Labs, Burlingame, CA, USA), rinsed with PBS, blocked with 10% normal serum, 1% Triton X-100 (Sigma-Aldrich, St. Louis, MO, USA) in PBS for 1 h. Primary antibodies were diluted in blocking solution as shown in Table 1 and were applied to sections for incubation overnight at room temperature. Secondary antibodies conjugated to Alexa-488, Alexa-568, or Alexa-647 (from Invitrogen (Carlsbad, CA, USA) or Jackson ImmunoResearch (West Grove, PA, USA)) were diluted to 10 μg/mL in blocking solution. Sections were rinsed three times in PBS, incubated with secondary antibody solution for 2 h at room temperature, rinsed three times in PBS, incubated with Hoechst 33342 (Invitrogen, 1/2000 in PBS with 0.05% Triton X-100), and rinsed twice in PBS. Coverslips were mounted with Fluoromount G (Southern Biotech, Birmingham, AL, USA). For CD3 labeling, the amount of Triton X-100 was decreased to 0.5% in the blocking solution and 0.2% in the antibody diluent.

Gastrocnemius muscles from the ipsilateral and contralateral sides were sectioned longitudinally at 25 μm. Twenty-four sections were obtained for each set (five sets/muscle, eight sections/slides, three slides total on each animal), and one set sections were chosen for neuromuscular junction (NMJ) and neurofilament (NF-H) staining, as described [28]. Briefly, sections were incubated in 0.1 M glycine in PBS, pH 7.3 for 30 min, blocked with 5% donkey serum (Jackson ImmunoResearch Laboratories), 0.5% Triton X-100, 1% BSA in PBS containing tetramethylrhodamine (TMR)—conjugated alpha-bungarotoxin (α-btx) for 1 h, permeablized with 100% methanol at −20 °C for 7 min, rinsed with PBS with 0.5% Triton X-100 (PBST), and incubated with primary antibody diluted in 1% BSA, 0.3% Triton X-100 in PBS at 4 °C for 48 h. After rinsing in PBST three times, sections were incubated at room temperature for 2 h with secondary antibody (donkey anti-chicken conjugated to Alexa Fluor^®^-488, 1/100, Jackson ImmunoResearch Laboratories, or donkey anti-rabbit conjugated to Alexa Fluor^®^-647 1/100, ab150075 Abcam) diluted in PBS. After rinsing in PBS three times, sections were coverslipped with Vectshield (H-1000, Vector Laboratories) and stored at 4 °C until being ready to image.

Paraformaldehyde fixed muscle sections were processed and embedded in paraffin according to routine histologic techniques. Sections, 5-µm thick, were stained with hematoxylin and eosin (H&E) stain according to standard methods, and examined by light microscopy.

### 2.10. Imaging and Image Processing

Fluorescent images were obtained at 10×, 20×, and 40× objective lenses with a laser scanning confocal microscope (Zeiss, Oberkochen, Germany, LSM710) equipped with 405, 488, 561, and 633 laser lines. Oil emersion was used with 40× objective. For images taken for pixel-based quantification, identical settings were used for all of the images in a set. For images chosen for publication, distracting artifacts were removed in ImageJ (version 1.38x, National Institutes of Health, Washington, DC, USA) [29] and levels were adjusted in Photoshop (version 13.0.6, Adobe Systems, San Jose, CA, USA) to maximize the signal-to-noise ratio so that the relevant features could be seen more clearly. For images chosen to highlight pixel-based quantification, sham- and ZIKV-group images were adjusted identically to enable equitable comparison.

### 2.11. Image Quantification

ImageJ was used for image quantification [29]. The cell counter plugin was used for manual cell counts. For pixel-based quantification of a given antibody signal, the area to be measured was defined by a region of interest (ROI), then thresholds of single channel images were adjusted to select pixels with signal above noise (positive pixels). Thresholds of all the images in a set were adjusted identically for equitable comparison. Then, positive pixels within the whole area ROI were measured to obtain the area occupied by positive pixels. The area of positive pixels was divided by the whole area of the main ROI to determine what proportion of the ROI was positive for the antibody. For co-localization analysis, pixels that were positive for two antibody signals were measured.

Spinal cord levels were identified using the mouse spinal cord atlas [30] and the location and morphology of clusters of choline acetyltransferase (ChAT) positive neurons. Because motor neurons for the gastrocnemius muscle are located at the L4–L5 level [31], sections from this level were chosen for analysis when possible. Eight to ten sections per animal were analyzed. ChAT+ neurons in the ventral horns were counted manually using the cell counter in ImageJ.

For the nerve cross sections, a stereological counting grid containing 25 × 25 μm^2^ boxes where each box was spaced 25 μm from the boxes above and below it, was placed over the nerve to obtain counts from a sample of the nerve, representing approximately one-fourth of the total area. An ROI was placed around the whole nerve and the counting grid was cut to eliminate all areas of the grid outside of the nerve. Stereological counting rules were used to count the number of myelinated axons, unmyelinated axons, degenerating axons (partially filled myelin sheaths), and empty myelin sheaths within the boxes of the cut grid. Images were converted to composite images so the neurofilament and myelin basic protein (MBP) channels could be toggled on and off during counting. All of the counts were done by a researcher who was blind to the status of each animal. The area of the cut grid, as well as the area of the whole ROI, were calculated and used to extrapolate the number of myelinated axons, unmyelinated axons, degenerating axons, and empty myelin sheaths within the whole area. Three sections per animal were analyzed.

For NMJ analysis, two sections per animal were analyzed. Confocal z-stacks (~1 μm step size) of two optimal fields of view containing NMJ endplates (α-btx+) and nerve fibers (NF-H+) were analyzed per section for a total of four fields of view per animal. NF-H ir, α-btx, and co-localization of NF-H and α-btx were quantified with pixel based-quantification as described above.

### 2.12. Statistics

Data were graphed and analyzed with Prism (version 7.0, GraphPad Software Inc., La Jolla, CA, USA) for statistical significance using *t*-tests or two-way ANOVAs with post-hoc *t*-tests. Linear regression was used for correlation analyses. Correlation significance was tested using the Spearman *r* test.

## 3. Results

The objective of this study was to determine the cause of hindlimb paralysis, so we investigated the involvement of the lumbosacral spinal cord, sciatic nerve, muscle, and neuromuscular junction. Motor deficits were quantitatively monitored with VPS assay.

### 3.1. Hindlimb Motor Deficits

**Figure 1:**
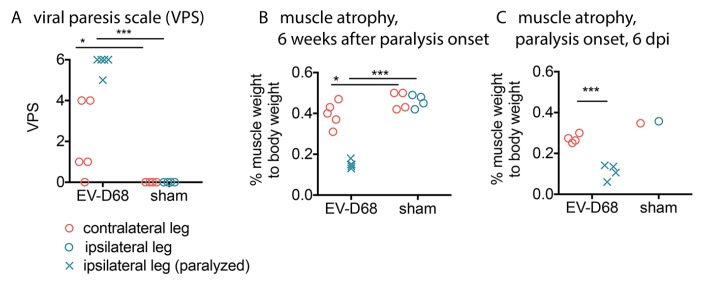
Hindlimb motor deficits and muscle atrophy in AG129 mice at onset of paralysis or 6 weeks after onset of viral paralysis. (**A**) Viral paresis scale (VPS). Weights of gastrocnemius and soleus muscles as a percentage of whole body weight were used to evaluate muscle atrophy measured at the time of necropsy at (**B**) 6 weeks after paralysis; and (**C**) at onset of paralysis. * *p* < 0.05, *** *p* < 0.001 as compared to respective leg controls using one-way analysis of variance.

At six weeks after onset of paralysis, all ipsilateral hindlimbs in relation to the side of i.p. viral injection was paralyzed (VPS of 5 or 6), and the values were statistically different from the sham-control ipsilateral hindlimbs (*p* < 0.001) (Figure 1A). Contralateral hindlimbs from infected mice did not have complete paralysis, but had increased VPS scores that were statistically different (*p* < 0.05) from the sham-control contralateral hindlimbs. These data suggested retrograde axonal transport of the virus to the spinal cord.

### 3.2. Muscle Atrophy

At time of necropsy immediately after performing the electrophysiological assays six weeks after paralysis onset, the gastrocnemius and soleus muscles were weighed and normalized to whole body weight (Figure 1B). Both ipsilateral and contralateral hindlimbs of infected mice had significantly lower muscle weights than the sham controls (*p* < 0.001 and *p* < 0.05, respectively). The infected mice had profound ipsilateral hindlimb atrophy and mild contralateral hindlimb atrophy.

### 3.3. Electrophysiology

**Figure 2:**
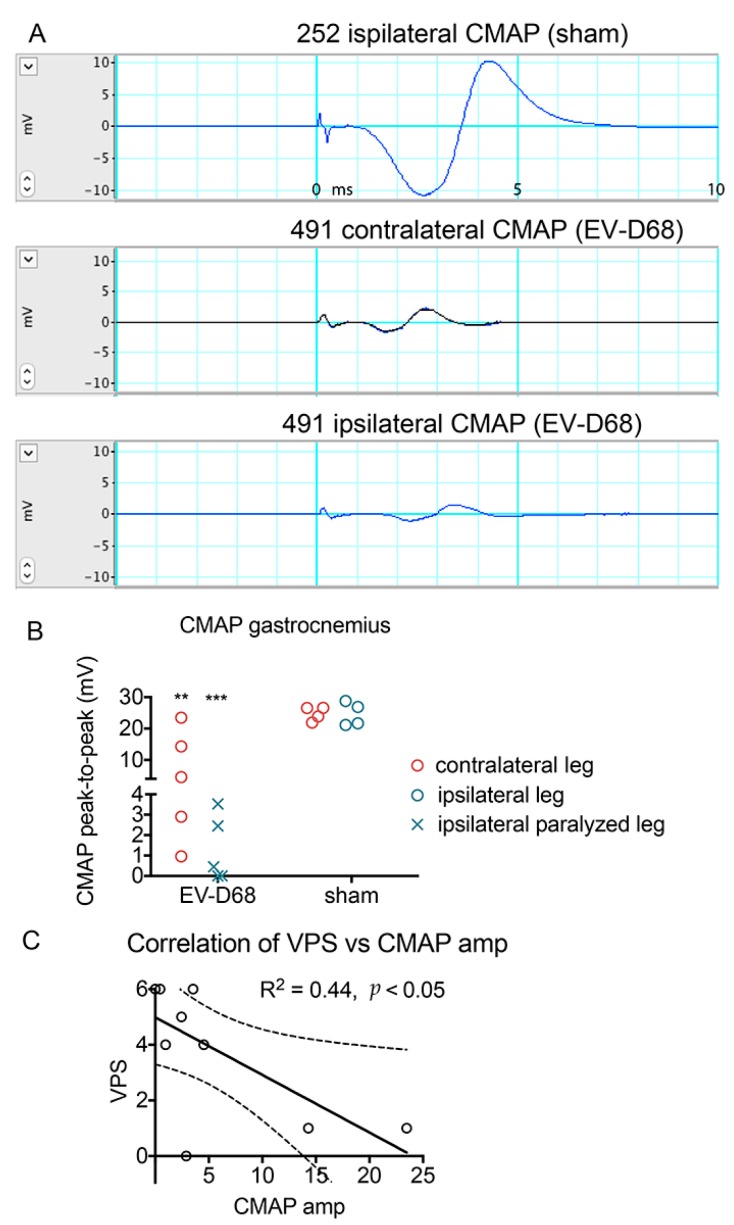
Deficits in Compound Muscle Action Potential (CMAP) amplitudes of EV-D68-infected AG129 mice 6 weeks after onset of paralysis. Blue and black lines are electrical potential tracings. (**A**) Examples of CMAP from sham-injected mouse (#252 ipsilateral), and from an EV-D68-injected mouse (#491) with no paralysis on the contralateral hindlimb and paralysis on the ipsilateral hindlimb; (**B**) CMAP amplitudes of gastrocnemius muscle of sham- and EV-D68-injected mice. Ipsilateral legs of infected mice were all paralyzed (“×” symbol). (**C**) Correlation of VPS and CMAP of infected mice only. Solid line represents linear regression analysis. Dotted lines represent 95% confidence interval. ** *p* < 0.01, *** *p* < 0.001 compared to respective sham controls using *t*-test.

Compound muscle action potential (CMAP) represents a synchronous activation of a group of motor neuron axons that are evoked by peripheral nerve electrical stimulation, and as such, can quantitatively evaluate the function of axons, neuromuscular junction (NMJ), muscle, and indirectly, motor neurons. In response to stimulation at the sciatic notch, amplitudes of CMAPs of both ipsilateral and contralateral sural muscles from infected mice were significantly reduced when compared to sham controls (*p* ≤ 0.001 and *p* ≤ 0.01, respectively) (Figure 2A,B). CMAP amplitude was negatively correlated with VPS score or motor deficit (Figure 2C). The correlation was R^2^ = 0.44 with a *p* ≤ 0.05, which indicated that CMAP amplitude was an indicator of motor function.

**Figure 3:**
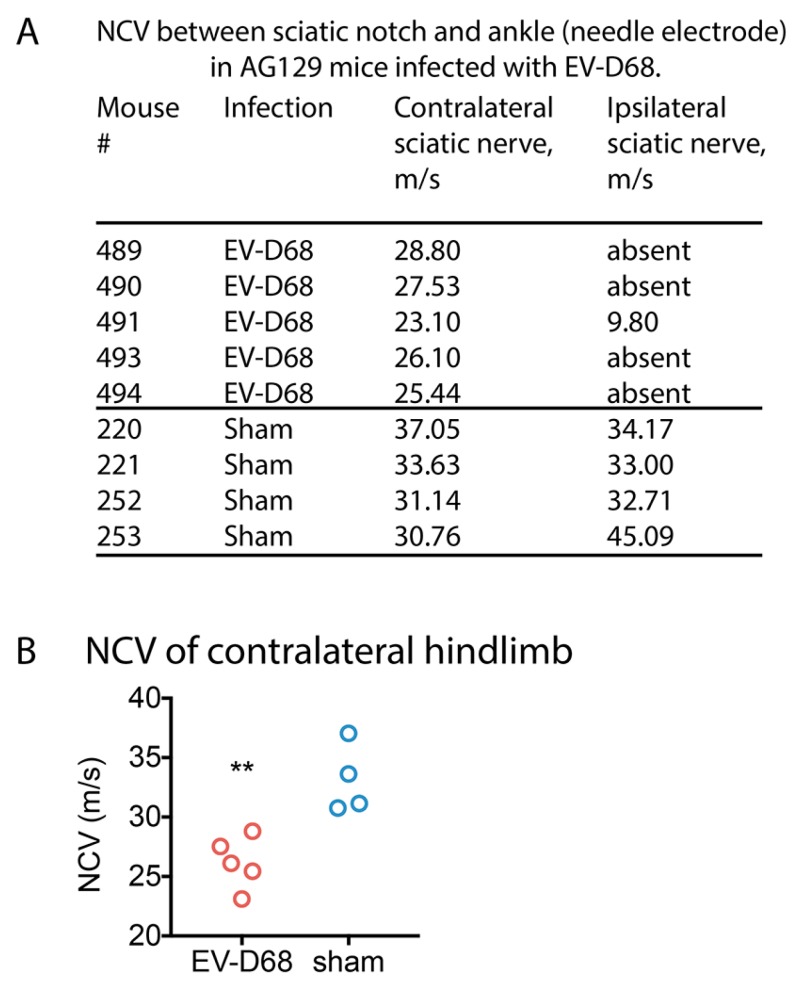
Reduced nerve conduction velocity (NCV) in AG129 mice infected with EV-D68 six weeks after onset of paralysis. (**A**) NCVs among EV-D68-infected and sham-infected mice; (**B**) NCV of contralateral EV-D68 (red circle) and sham (blue circle) hindlimbs. ** *p* < 0.01 using *t*-test, # mouse ID number.

To evaluate nerve conduction velocity (NCV) of the sciatic and tibial nerves, NCV was calculated after stimulation at the sciatic notch and the ankle and recording CMAPs at the plantar foot muscle. The NCV was reduced in virally infected mice when compared to controls (Figure 3). The NCV of the ipsilateral paralyzed hindlimb of one EV-D68-infected mouse (#491) was 9.8 m/s as compared to values above 30 m/s of sham-infected mice (Figure 3A). Ipsilateral CMAPs were not detected with either proximal or distal recording electrodes of the other four ipsilateral limbs, so NCV could not be calculated. The NCVs of contralateral hindlimbs of infected mice could be recorded and were calculated to be statistically lower as compared to contralateral limbs of sham-infected mice (*p* < 0.01) (Figure 3B).

**Figure 4:**
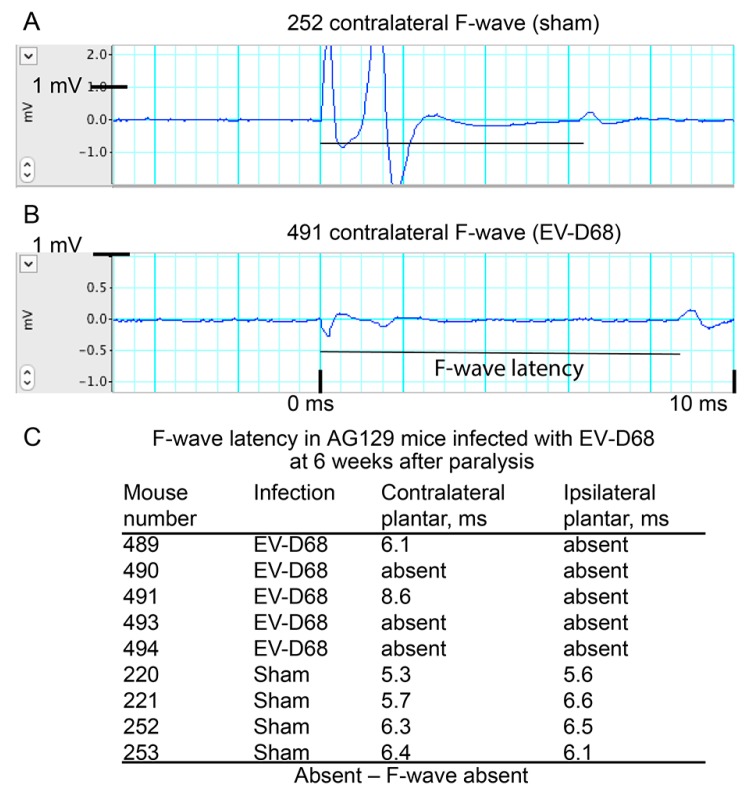
F-wave tracings and latency stimulated at popliteal nerve behind knee and recorded at plantar muscles from AG129 mice infected with EV-D68 six weeks after onset of paralysis. (**A**) F-wave of contralateral hindlimb of #252 sham mouse; (**B**) F-wave of contralateral hindlimb of #491 EV-D68-infected mouse; (**C**) F-wave latencies among EV-D68-infected and sham-infected mice.

F-wave tracings were detected; however, we do acknowledge that the tracings could be H-reflexes. Nevertheless, both signals go through alpha motor neurons. Examples of tracings from EV-D68-infected and sham-infected mice are shown (Figure 4A,B, respectively). F-waves could only be detected in 2 of 5 contralateral limbs of infected mice and none of the ipsilateral limbs. F-waves could be detected in all the limbs of sham-infected mice (Figure 4B). This suggests that the neurons were damaged, because the F-waves were undetectable, and the M-waves were present. An F-wave latency of 8.6 m/s of a contralateral limb from an infected mouse (#491) was considerably greater than the latencies <6.4 m/s of the sham-infected mice. The other measurable contralateral latency of 6.1 m/s from an infected mouse (#489) was within sham-infected ranges. These data indicate that motor neurons or axons may be impaired, which is consistent with data of CMAP amplitudes and NCV.

**Figure 5:**
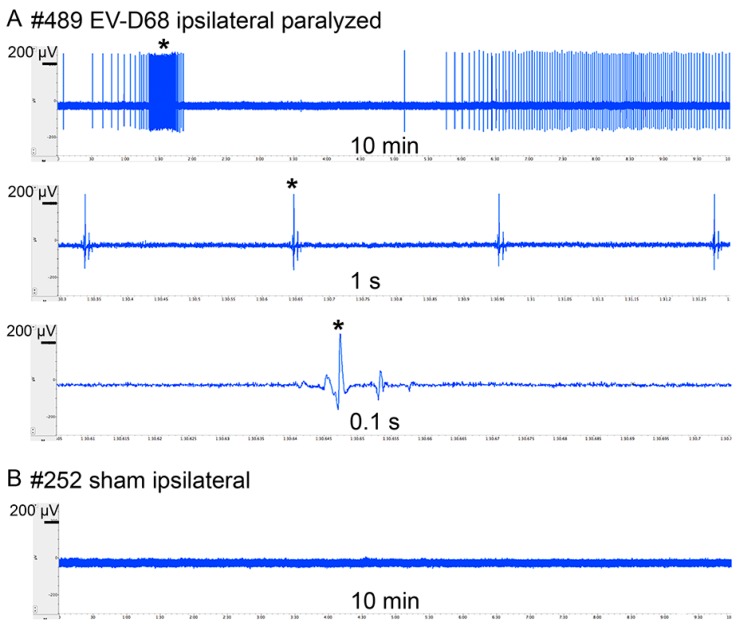

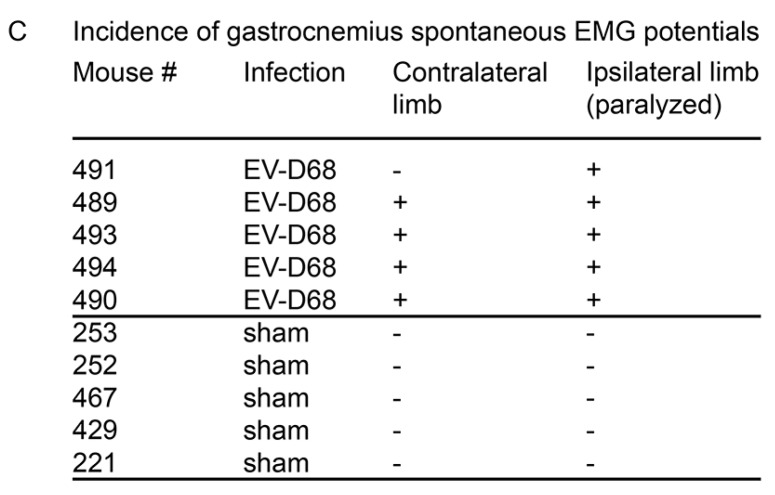
Spontaneous electromyographic (EMG) fibrillation potentials of gastrocnemius muscle from AG129 mice infected with EV-D68 six weeks after onset of paralysis. * indicates same time reference between tracings. (**A**) Paralyzed ipsilateral hindlimb of #489 infected with EV-D68 had fibrillation; (**B**) Ipsilateral hindlimb of #252 sham mouse did not have fibrillation; (**C**) Incidence of fibrillation among EV-D68-infected and sham-infected mice. + symbol indicates presence of spontaneous EMG. − symbol indicates absence of spontaneous EMG.

Fibrillation potentials occur when muscle fibers lose contact with the innervating axons, thereby producing spontaneous action potentials. Since data indicate extreme gastrocnemius atrophy and probable denervation of muscles, we measured spontaneous electromyographic (EMG) potentials in the gastrocnemius (Figure 5). The tracings (S1 spontaneous EMG tracings) were also converted to audio tracks for diagnostic analysis (S2–S5 spontaneous EMG audio). The paralyzed ipsilateral limbs of all five isoflurane-anesthetized EV-D68-infected mice had spontaneous potentials, which contained tracings characteristic of fibrillations and perhaps fasciculations. Ninety percent of the non-paralyzed contralateral hindlimbs from infected mice had identifiable spontaneous potentials. None of the limbs from sham-infected mice had any spontaneous potentials. These data suggest axonal dysfunction or denervation of muscle fibers.

### 3.4. Spinal Cord Immunohistochemistry (IHC)

**Figure 6:**
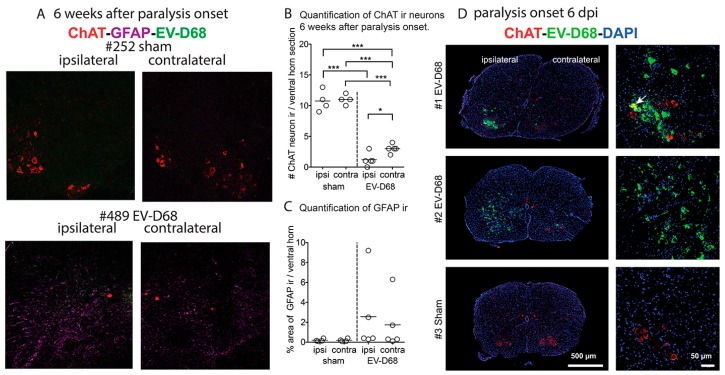
Reduced motor neurons in lumbosacral spinal cords of AG129 mice. (**A**) ChAT ir, GFAP ir, and EV-D68 ir in lumbosacral spinal cords of #252 sham- and #489 EV-D68-infected mice on ipsilateral and contralateral sides (infected i.p. 10 dpi, 6 weeks after paralysis onset); (**B**) Quantification of ChAT ir neurons in (**A**,**C**); Quantification of GFAP ir in (**A**,**D**); ChAT ir, EV-D68 ir, DAPI in lumbosacral spinal cord at paralysis onset challenged (six days post-viral injection, dpi) in (**A**). Arrow indicates co-localized EV-D68 ir and ChAT ir. *** *p* < 0.001 using one-way ANOVA. * *p* < 0.05 when using *t* test, but not significant when using one-way ANOVA. Vertical dotted lines separate sham and infected data.

Due to reduced CMAP and the absence F-waves that implicate dysfunction or death of motor neurons, ChAT immunoreactive (ir) motor neurons were quantified in mice challenged i.p. with the virus. The numbers of ChAT ir neurons from infected mice in either the contralateral or ipsilateral sides of the spinal cord were statistically different from the corresponding limbs of sham-infected mice (*p* < 0.001) (Figure 6A,B). The number of ChAT ir cells in the ipsilateral side of the cord from infected mice was statistically lower than the cells on the contralateral side (*p* < 0.05). GFAP ir was increased in some, but not all, infected mice at this time point (Figure 6C). No EV-D68 ir was observed in cords of these mice (Figure 6A). Nevertheless, EV-D68 ir was observed in spinal cords of i.p. infected mice six days post-injection (dpi) at the time of paralysis onset (Figure 6D). Therefore, the virus was presumably cleared within six weeks after onset of paralysis. These data implicate the loss of motor neurons as the cause of paralysis. Also, the numbers of motor neurons between the contralateral or ipsilateral sides might represent a threshold for loss of motor neurons necessary for complete paralysis to develop, because the ipsilateral limbs were severely paralyzed whereas deficits in the contralateral limbs were less severe.

### 3.5. Sciatic Nerve IHC

**Figure 7:**
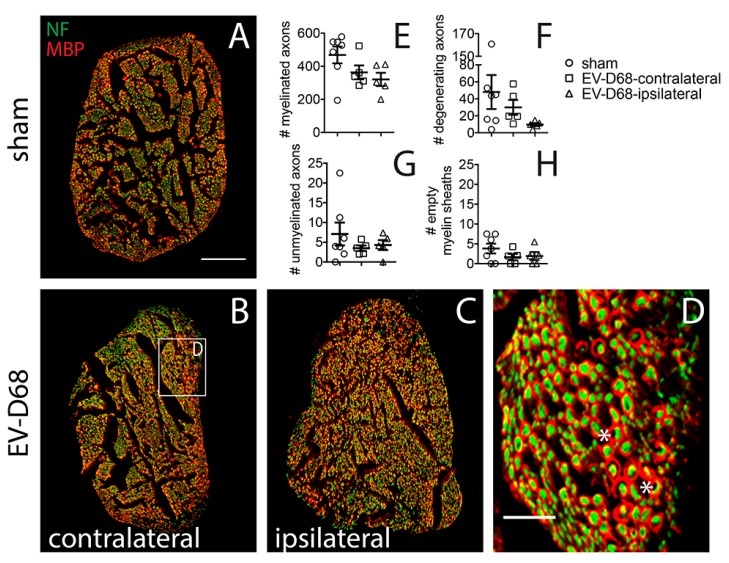
Myelin basic protein (MBP) ir and NF ir in sciatic nerves of EV-D68-infected mice 6 weeks after paralysis onset compared to sham. (**A**) sham; (**B**) contralateral EV-D68 sciatic nerve; (**C**) ipsilateral EV-D68 sciatic nerve; (**D**) enlargement of boxed area in B showing myelin sheaths containing degenerating axons (asterisk); (**E**) # myelinated axons; (**F**) # degenerating axons; (**G**) # unmyelinated axons; (**H**) # empty myelin sheaths. No statistical differences between groups. Scale bars = 100 µm in (**A**) (**A**–**C** are same scale); 25 µm in (**D**).

Myelin basic protein (MBP) ir and neurofilament ir were examined in all the EV-D68- and sham-infected mice (Figure 7A–C). There were no statistically significant morphological changes to myelinated (Figure 7E), degenerated axons (Figure 7F), unmyelinated (Figure 7G), or empty myelin sheaths (Figure 7H). Even though there were some trending differences between the groups, there was no indication that the ipsilateral nerves had abnormal nerve morphology.

**Figure 8:**
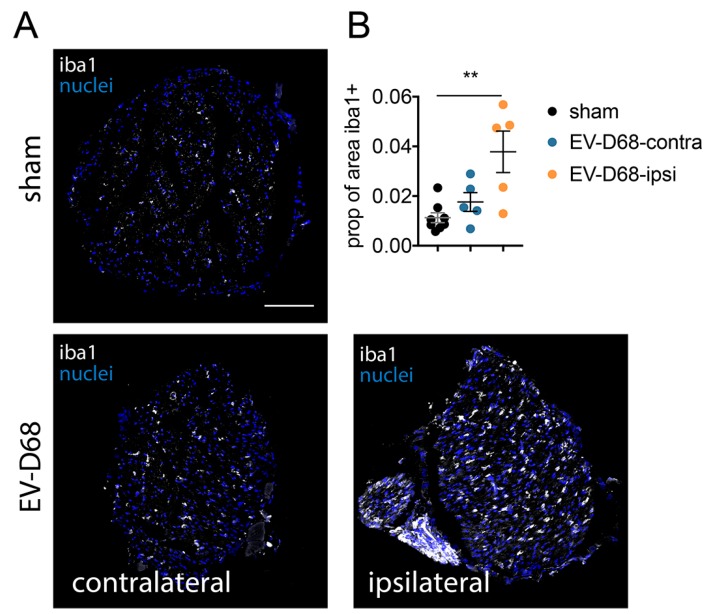
Increased macrophage/microglial iba1 ir in sciatic nerves of EV-D68-infected mice six weeks after paralysis onset compared to sham. (**A**) Iba1 ir in coronal cross-sections of sciatic nerves (100 µm bar); (**B**) Quantification of iba1 ir. ** *p* < 0.01 using one-way analysis of variance.

The macrophage/microglial marker, iba1 ir; neutrophil marker, Ly6G; and T cell marker, CD3, were assayed as markers for inflammation (Figure 8). Of the three markers, only iba1 ir was statistically different from sham-infected control samples at six weeks of paralysis. The sciatic nerve on the ipsilateral side had a mean iba1 ir value of greater than three-fold of sham-infected mice (*p* < 0.01) and <2-fold of the contralateral side. These data suggest that inflammatory responses of the sciatic nerve during this later stage of paralysis were mild as observed with increases of iba1 ir, but not CD3 ir or Ly6G ir.

### 3.6. Muscle

**Figure 9:**
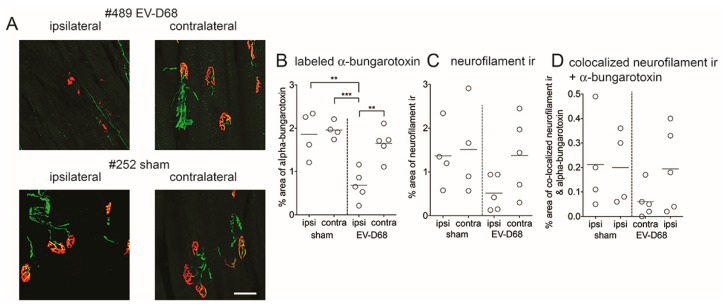
Reduced neuromuscular junction (NMJ) innervation in gastrocnemius muscles of AG129 mice infected with EV-D68 six weeks after paralysis onset. (**A**) neurofilament (NF) ir (green), fluorescence-tagged α-btx (red) in sham- and EV-D68-infected mice. Scale bar = 50 µm; (**B**) Quantification of labeled α-btx to detect motor plates; (**C**) Quantification of neurofilament ir to detect axons; (**D**) Quantification of co-localization of neurofilament ir and labeled α-btx to detect innervated NMJ (yellow). Vertical dotted lines separate sham and infected data. ** *p* < 0.01, *** *p* < 0.001 using *t*-test.

The observation of reduced spontaneous EMG potentials in muscles of i.p.-infected-mice suggested that denervation of muscles had occurred, and muscle atrophy may have been a consequence of denervation. To directly analyze denervation of muscle at six weeks of paralysis, innervated synapses were identified with labeled α-bungarotoxin (α-btx, post-synaptic motor endplates) and anti-neurofilament (NF) antibody [32] (Figure 9A). Gastrocnemius motor endplates (α-btx-labeling) of ipsilateral limbs of viral infected mice were statistically less than labeling of sham-infected mice (Figure 9B). NF ir (Figure 9C) and adjacent NF ir + α-btx (Figure 9D) were less on the ipsilateral side compared to sham-infected values, but not at a statistically significant level.

**Figure 10:**
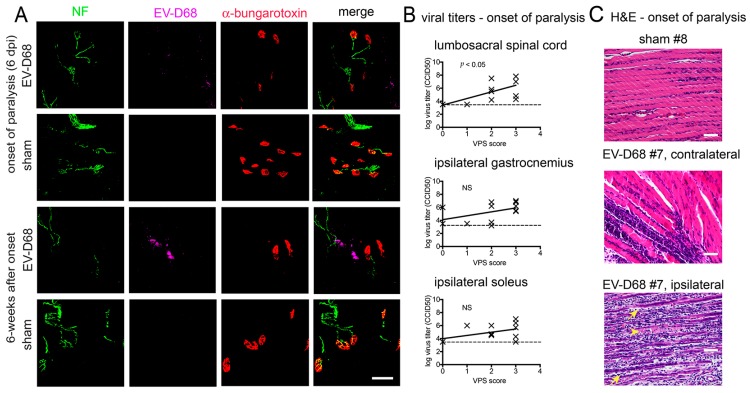
Reduced neuromuscular junction (NMJ) innervation in gastrocnemius muscles of AG129 mice infected with EV-D68. (**A**) neurofilament (NF) ir, EV-D68 ir, fluorescence-tagged α-btx in sham- and EV-D68-infected mice at onset of paralysis (6 days after viral challenge) and at six weeks after paralysis onset; (**B**) EV-D68 infectious viral titer (50% cell culture infectious dose, CCID50) versus motor score at onset of paralysis at 7–8 dpi; (**C**) Hematoxylin-eosin (H&E) staining of gastrocnemius muscle, image of sham #8 and image of contralateral EV-D68 #7, image of ipsilateral EV-D68 #7 at onset of paralysis at 7–8 dpi. Necrotic myofiber (yellow arrow head). Regenerating myofiber (yellow arrow). 50 µm for all bars. Dotted line indicates limit of detection.

Infection, denervation, and atrophy of muscles at six weeks of paralysis raised questions about the status of muscles at onset of paralysis at 6–8 days after viral challenge. Like muscle at six weeks of paralysis, EV-D68 ir and infectious virus was present in ipsilateral muscles at the onset of paralysis (Figure 10A,B). EV-D68 ir (Figure 10A) and infectious virus was also identified in the spinal cord (Figure 10B), unlike the lack of viral detection in the spinal cord at six weeks after onset of paralysis. Muscle atrophy was also apparent at onset of paralysis (*p* < 0.001) (Figure 1C), which we considered to be remarkable at this early stage of paralysis. These data suggested that muscle atrophy was not exclusively due to a slower process of denervation caused by motor neuron death, and that pathogenesis may have occurred directly in muscle tissues at this early onset-stage of paralysis to contribute to motor deficits.

To investigate this postulate further, hematoxylin-eosin (H&E) histological analysis was performed, which revealed that ipsilateral gastrocnemius muscle at 8 dpi just after the onset of paralysis had severe multifocal to coalescing neutrophilic and histiocytic myositis with prominent early myocyte regeneration. Images in Figure 10C shows variability of pathology in two images within a single muscle of the same animal. The contralateral muscles had mild to severe myositis. In muscles with severe inflammation, there was an apparent loss of myocytes with frequent early regenerating myofibers (Figure 10C). In muscles with mild inflammation, mild myofiber degeneration was apparent, but no regeneration was observed. These data suggested that myocyte degeneration was occurring in ipsilateral muscles, but was rebounding with some regeneration. The infection and pathogenesis of the contralateral sides were probably delayed when compared to the ipsilateral side.

Correlation analysis of motor functions was done with viral titers in the spinal cord and muscle tissues (Figure 10B). Viral titers in the lumbosacral spinal cord was correlated with the severity of motor deficits (*p* < 0.05), but no correlation was observed with muscle tissues when compared to motor function. These data suggested that spinal cord involvement contributed to motor deficits, but one should not dismiss that the observed myositis might also contribute to motor deficits.

### 3.7. Intranasal EV-D68 Viral Challenge 

**Figure 11:**
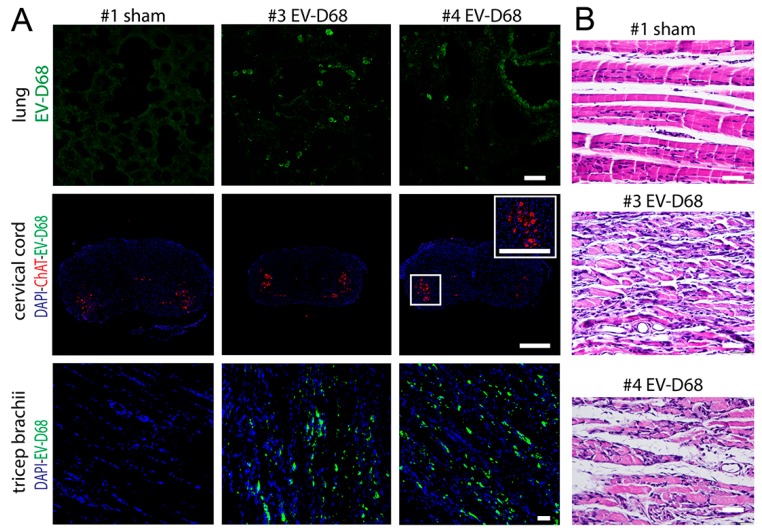
EV-D68 ir in muscle only with myositis and myofiber degeneration in AG129 mice challenged intranasally at 5 days of age and assayed at peak paralysis 8 days later. (**A**) EV-D68 ir in lung, ChAT ir and EV-D68 ir in cervical cord and EV-D68 ir in tricep brachii; (**B**) Hematoxylin-eosin (H&E) staining of bicep muscle in #1 sham, #3 EV-D68, and #4 EV-D68 AG129 mice at onset of paralysis eight days after viral challenge. 500 µm bar for cervical cord in (**A**). All other bars are 50 µm.

Since EV-D68 is a respiratory virus unlike other enteroviruses, we exposed four five-day-old AG129 mice by intranasal installation (both nares) of the mouse-adapted virus. EV-D68 ir was present in lungs of infected mice, but not sham-mice (Figure 11A). Like i.p. challenge, EV-D68 ir was readily apparent in muscle tissue. Unlike i.p. challenge, the spinal cord was not infected. The biceps muscle of paralyzes forelimbs from intranasally challenged mice had neutrophilic myositis with severe myofiber degeneration and necrosis. (Figure 11B). Since the VPS assay measures hindlimb deficits, we only qualitatively observed the animals for overt paralysis, because VPS could not be performed. Eight days after exposure, 2 of 4 mice developed paralysis of either right or left forelimbs (S6 video forelimb paralysis). Unlike i.p. challenge, paralysis developed on either side. When paralyzed, the mice were euthanized, and lung and forelimb muscle (triceps brachii and biceps) from limbs were prepared for IHC and H&E staining.

## 4. Discussion

### 4.1. Unique Contributions

The contributions of these studies are that clinical electrophysiological examinations complemented with IHC demonstrated that EV-D68 clade B1 causes acute flaccid paralysis, myelitis, myositis, and muscle atrophy at onset of paralysis, and six weeks after onset in AG129 mice challenged i.p. with virus at 10 days of age. The data suggests that motor deficits are caused by loss of motor neurons in the spinal cord, but early muscle infection and disease may also contribute to deficits. Additionally, infection of the spinal cord likely involves retrograde axonal transport, because spinal cord infection is greatest on the ipsilateral side, and paralysis develops in the ipsilateral limbs relative to the side of viral injection.

To model more natural respiratory infection, mice at five-days-old challenged by intranasal installation with EV-D68, adapted for propagation in mouse lung developed forelimb paralysis eight days later. The mice had infection in forelimb muscles with associated myositis, but not in the cervical spinal cord (Table 2). These data suggest that human respiratory infection may also result in myositis and paralysis without spinal cord infection.

### 4.2. Myelitis in Intraperitoneally Challenged Mice

The prototypic enterovirus, poliovirus, and other enteroviruses like EV-71 cause myelitis and destruction of motor neurons [33]. Data from mice injected i.p. with EV-D68 of this study is diagnostic of poliovirus-like myelitis and acute flaccid paralysis. The F-wave relaying through the motor neuron could not be detected in paralyzed limbs, likely because EV-D68 destroyed motor neurons. Indeed, immunofluorescence analysis revealed that motor neurons were depleted in the ipsilateral ventral horn of the spinal cord at six weeks after viral challenge (~7-week old). Even though viral immunoreactivity could not be detected in the spinal cord 6 weeks after onset, it was present at onset of paralysis eight days after viral challenge. In samples from mice at early onset of paralysis, EV-D68 ir was co-localized with ChAT ir, indicating direct infection of motor neurons by the virus, which probably led to the death and depletions of motor neurons during the five-week period of paralysis.

To investigate peripheral neuropathy as a possible cause of paralysis, we examined the morphology of sciatic nerves. Microscopically, the integrity of the myelinated axons did not appear to be worsened by the virus. Cellular inflammation was mild due to increases in only macrophage/microglial cells, but not in T cells or neutrophils. Yet, all five infected mice had hindlimb paralysis in the ipsilateral leg at the time of histological examination. These data suggested that peripheral neuropathy was not the cause of paralysis.

### 4.3. Muscle Disease in Intraperitoneally Challenged Mice

To investigate the role of muscles in paralysis of mice challenged i.p. with EV-D68, we quantified the motor plates, neuromuscular junctions (NMJs), and innervating axons of the motor plates after six weeks of paralysis. In paralyzed limbs, these motor structures were decreased, which led to the postulate that death of neurons caused denervation of limb muscles. Unexpectedly, muscle infection and myositis occurred as early as onset of paralysis 6–8 days after viral challenge, which was probably not enough time for death of motor neurons to cause denervation and muscle atrophy. Viral infection of muscle caused severe inflammation and myofiber degeneration, which was most pronounced in ipsilateral limbs. Therefore, muscle disease, in addition to myelitis, could have contributed to paralysis in these i.p. challenged mice.

Although enteroviruses are not typically associated with skeletal myositis, coxsackievirus B3, and to a lesser degree, other enteroviruses, such as enterovirus 71 (EV71) [34] and echovirus [35], cause myocarditis. Some evidence, however, suggests that non-polio enteroviruses can be associated with myositis. In a molecular epidemiological study, non-polio enteroviruses (71.4% EV-A, 18.4% EV-B) identified in acute flaccid paralysis cases in China revealed that 13% of febrile patients had myositis [36]. Mouse studies definitively demonstrate causation of skeletal myositis by enterovirus. Mouse-adapted EV71 caused skeletal necrotizing myositis, paralysis, and death in 2-week-old mice. No lesions of the central nervous system could be identified in these mice [37]. In another mouse study [38], skeletal muscle was the primary site of replication in neonatal mice with paralysis, yet the central nervous system was not infected.

Muscle infection, however, may have been specific for the mouse as compared to the humans for various reasons. Murine myocytes may support EV infection and replication more readily than in human cells. Although we did not do a comparison of human-to-mouse cell susceptibility, we do propogate EV-D68 in RD cells, which is a human rhabdosarcoma muscle cell line. Another reason for myositis in mice, is that mice are injected parenterally with the EV-71 where the virus might have greater access to myocytes, unlike natural airborne transmission with EV-D68 or fecal-oral transmission with EV-71. Another reason might be that the diagnosis of myositis is overlooked in the presence of profound respiratory infection and suspected CNS disease with paralysis.

If the assumption is correct that EV-D68 can cause some degree of myositis in human patients, the resulting myositis might contribute to muscle weakness perhaps with or without spinal cord disease. This might help explain why EV-D68 is usually undetectable in CSF of patients with AFM. When mice were challenged i.p., EV-D68 ir was readily observed in the spinal cord at the early onset of paralysis, but is absent after weeks of paralysis. Yet, EV-D68 ir is present in muscle at onset and persists six weeks after the onset of paralysis. Future studies with the paralysis mouse models of this study should track EV-D68 in the CSF and muscle enzymes over time. Perhaps muscle disease plays a larger role in paralysis than previously anticipated, such that the detection of EV-D68 in the CSF may not be dependable marker for paralysis.

Muscle atrophy in the human disease is significant in the weeks to months following onset of muscle weakness, which may be caused by denervation of muscles due to destruction of motor neurons and axons [5], or as discussed above, viral induced myositis. We also observed significant muscle atrophy in mice challenged i.p. at onset of paralysis and after six weeks of paralysis. Immunofluorescence analysis at six weeks of paralysis, revealed that infected mice had significant loss of NMJ innervation, which is consistent with the hypothesis that muscles are being denervated due to the destruction of motor neurons and axons. However, at onset of paralysis (day 8 after viral challenge), skeletal muscle was infected with the virus and mice had severe multifocal to coalescing neutrophilic and histiocytic myositis. There was an apparent loss of myocytes with frequent early regenerating myofibers. Our interpretation of these data is that infection and early myositis caused muscle damage. Motor neurons were depleted by five weeks of paralysis, so denervation that is initiated by loss of motor neurons may compound the neurological function of the muscles. One should also consider that axonal sprouting from surviving motor neurons and further regeneration of muscle fibers may also occur over time.

Long-term studies of paralyzed mice may indicate if muscle fibers can regenerate, if axonal sprouting can improve motor functions, and how many surviving motor neurons are required for functional recovery. Such studies could be clinically relevant because recoveries of patients are variable [9], which may reflect variable axonal sprouting. For example, 5% of 56 AFM patients reported complete recovery. Eighteen percent reported full functionality at 4.2 months (median) after onset [10]. Long-term studies with these mice might provide answers about the level of muscle regeneration, neuron loss, axonal sprouting, and renervation of NMJ, and ultimately, strategies for therapeutic interventions. Electrophysiologically, fibrillation potentials would be expected to abate and CMAP amplitudes may increase along with the appearance of polyphasic muscle action potentials if chronic re-innervation were occurring [5]. Another aspect to be investigated is if the virus persists in muscle because, after six weeks of paralysis, the virus was nearly absent from the spinal cord, but still present in muscle tissue.

### 4.4. Muscle Disease in Intranasally Challenged Mice

To best model natural human EV-D68 infection, mice were challenged intranasally with EV-D68. Since EV-D68 in human patients causes severe respiratory infection, we reasoned that we needed to use virus passaged in mice by intranasal installation that would greatly favor lung infection. Indeed, lung titers were 10-fold greater in mouse lung using this adapted virus as compared to the wild-type virus (manuscript in preparation).

Two of four five-day-old AG129 mice challenged intranasally with adapted EV-D68 developed unilateral paralysis of either forelimbs eight days after viral challenge. Interestingly, no EV-D68 ir was observed in the spinal cord at peak paralysis, yet, EV-D68 ir and myositis was readily observed in forelimb triceps muscles. These data suggested that myositis caused paralysis during lung infection, not myelitis.

### 4.5. Electrophysiological Interpretation in Intraperitoneally Challenged Mice

In mice challenged i.p., CMAP and NCV data are interpretable in the context of loss of motor neurons, loss of NMJs, presence of myositis, and lack of robust peripheral neuritis. With the death of motor neurons, the electrochemical function of axons probably began to diminish over time through the five-week period of paralysis. Consequently, the amplitudes of CMAPs stimulated from the sciatic notch and recorded on the gastrocnemius were dramatically diminished in ipsilateral paralyzed limbs, and to a less extent, in the contralateral limbs. M-waves used to calculate NCV were detectable in both proximal and distal EMG anatomical sites in contralateral (left) non-paralyzed limbs, but not in the ipsilateral paralyzed limbs, because nerve functions were likely degenerated enough to prevent recording of NCV in ipsilateral paralyzed limbs. In contralateral limbs, NCV was increased when compared to values of sham-infected mice, which is probably due to neuron cell body damage and subsequent declining nerve function.

In human EV-D68 patients, CMAP amplitudes are variable within the first three weeks, but reduced amplitudes are consistently observed after three weeks of onset of AFM. Fibrillations potentials are not detected within the first week, but develop in subsequent weeks. EMG changes persist for months and in some cases over a year. Additionally, motor deficits are asymmetrical [9]. These disease phenotypes are consistent with those that are observed in the EV-D68 model described herein, i.e., asymmetric reduced CMAPs and spontaneous EMG potentials. Moreover, EMG changes could be seen in contralateral mouse limbs without paralysis, which is similar to EMG changes that are observed in non-paralyzed limbs of human patients [5,9].

### 4.6. Biological Markers of Causation

The data of this report and experiments with neonatal mice [20] prove that EV-D68 can cause AFM in mice. Nevertheless, the data only suggest a possible causation of AFM in human subjects. Future studies with these mouse models could answer important questions about etiology and pathogenesis of human EV-D68 disease. Lack of detection of infectious virus or viral RNA in human CSF suggests that EV-D68 is not the causation of AFM. However, this may not be surprising since the incidence of detecting known neurotropic enteroviruses, such as enterovirus 71 and poliovirus in the CSF is between 0–5% [39,40]. Time-dependent studies could be performed to determine if or when virus or anti-EV-D68 IgM antibodies can be detected in the CSF, and if pleocytosis with lymphocytic predominance, and elevated protein without glucose is present in the CSF, as is found in EV-D68-associated AFM [5,9,10]. Also, when considering the presence of myositis in mice challenged either i.p. or intranasally, muscle enzymes may also be biological markers of causation.

### 4.7. Retrograde Transmission

Retrograde transmission of the virus into the spinal cord may occur in the mouse model reported herein and in neonatal mouse model [20], because unilateral viral injection causes paralysis of ipsilateral limbs. If retrograde transmission does occur, retrograde infection from lungs to the cervical cords could explain asymmetric paralysis of forelimbs observed in human patients with EV-D68-respiratory infections [5,10,12]. Involvement of upper extremities and proximal muscle groups of C5 to C6 are more affected than distal muscle groups with EV-D68-associated AFM cases. Many AFM patients during the acute phase are hospitalized, and some require ventilatory support due to bulbar involvement and an inability to protect the airway. Respiratory failure can occur due to paralysis of respiratory musculature. Upper cranial motor, and bowel and bladder dysfunctions can also occur [5,9,10]. Since retrograde infection appears to occur in these mice, cervical spinal cord or bulbar infections might be favored with injection of the virus in forelimbs or upper torsos.

Likewise, poliovirus (PV), which is another neurotropic enterovirus, injected in the calf muscle of human poliovirus receptor (hPCR) transgenic mice causes hindlimb paralysis first in the ipsilateral side by entry into the sciatic nerve [41]. Another investigation of hPVR mice reveals, however, that axonal pathway from muscle is not primarily involved with entry of the virus into the CNS when the virus is injected intravenously [42]. The role of axonal-CNS infection with poliovirus is uncertain, and likewise, the clinical importance of axonal infection of the CNS with EV-D68 is yet to be determined.

### 4.8. Pediatric Disease

The use of 5- and 10-day old mice of this study models EV-D68 incidence of disease in children (mean 7.1 years old) as compared to adults where only 15% of cases are over 21 years of age [9]. By using mouse body life span, age of adulthood, sexual maturity, weight, weight of eye lens, epiphyseal closure, tooth wear, and lifespan, investigators predicted that the number of mouse days equal to one human year [43]. Using onset of adulthood, 2.6 mouse days would be equal to 1 human year; therefore, mice that are used in this study would be analogous to human ages between 2- to 4-years-old. Besides using ages of mice appropriate for modeling pediatric disease, the mice of this study are larger for performing clinically relevant electrophyiological tests as correlates to human disease.

## 5. Conclusions

Intraperitoneally viral challenge caused paralysis in mice by infection of the spinal cord and skeletal muscle, leading to myelitis and destruction of motor neurons, in addition to myositis and myofiber degeneration. In mice challenged intranasally with the virus, only myositis was associated with paralysis. Causation of paralysis by muscle disease may help to resolve the controversy that the virus can cause paralysis, even if it cannot be identified in CSF. These models will have future applications for investigating biomarkers for causation, time-course of diagnostic electrophysiology, histopathology, axonal sprouting, renervation for recovery, and determinants of disease in neonatal, juvenile, and adult mice.

## Figures and Tables

**Table 1:** Primary Antibodies.

Antibody Specificity	Antibody Type	Company, Catalogue Number	Dilution	Marker
ChAT	goat pAb	Millipore (Burlington, MA, USA), AB144P	1/100	motor neurons
VP2	rabbit pAb	GeneTex (Irvine, CA, USA), GTX132314	1/100	EV-D68
iba1	goat pAb	Abcam (Cambridge, MA, USA), ab5076	1/200	macrophage/microglia
GFAP	rat IgG2a-kappa mAb	Invitrogen (Carlsbad, CA, USA), 13-0300	1/500	astrocytes
NF-H	chick pAb	Aves Labs (Tigard, OR, USA), NFH	1/200	axons
MBP	rat IgG2a mAb	Abcam, ab7349	1/200	axon sheath
α-bungarotoxin-TMR		Biotium (Fremont, CA, USA), 00012/00014	1/500	neuromuscular junction

Abbreviations: pAb, polyclonal antibody; mAb, monoclonal antibody.

**Table 2:** Comparison of intranasal vs. intraperitoneal EV-D68 challenges.

		Intraperitoneal Viral Challenge at 10-Day-Old)		Intranasal Viral Challenge at 5-Day-Old)
		At onset of paralysis	After 6 weeks of paralysis	
EV-D68 ir ^a^	Muscle	+	+	+
	Cord ^b^	+	−	−
Histopathology ^c^	Muscle	+	+	+
	Cord	+	+	−
Limb paralysis		Ipsilateral hindlimb	Ipsilateral hindlimb	Both forelimbs

^a^ Presence of EV-D68 immunoreactivity. ^b^ Lumbosacral spinal cord. ^c^ Histopathology as seen in H&E sections or with presence of immune cells with confocal microscopy. + indicates paralysis, − indicates absence of paralysis.

## Supplementary Materials

The following are available online at www.mdpi.com/1999-4915/10/1/33/s1. S1 spontaneous EMG tracings. Spontaneous EMG tracings of gastrocnemius muscles of EV-D68-infected mice #489, #491, #493, and #494. Right limb are ipsilateral and left limb are contralateral. Single capitalized letters indication a specific location in the tracing and correlate with the audio tracks of S2 supporting information. S2 spontaneous EMG audio #489. Examples of audio tracks of spontaneous EMG tracings of ipsilateral #489 of B in S1 supporting information. S3 spontaneous EMG audio #491. Examples of audio tracks of spontaneous EMG tracings of ipsilateral #491 of D in S1 supporting information. S4 spontaneous EMG audio #491. Examples of audio tracks of spontaneous EMG tracings of ipsilateral #491 of C in S1 supporting information. S5 spontaneous EMG audio #494. Examples of audio tracks of spontaneous EMG tracings of ipsilateral #494 of D in S1 supporting information. S6 video forelimb paralysis. Video of forelimb-paralyzed mouse.
