# Supplementary material for: Causation of Acute Flaccid Paralysis by Myelitis and Myositis in Enterovirus-D68 Infected Mice Deficient in Interferon αβ/γ Receptor Deficient Mice
Source: Viruses. 2018 Jan 12;10(1):33. doi: 10.3390/v10010033 (PMC5795446; doi:10.3390/v10010033)
Supplement: Supplementary file 1 [file viruses-10-00033-s001.zip › Supplementary/S1 tracings (1).pdf]

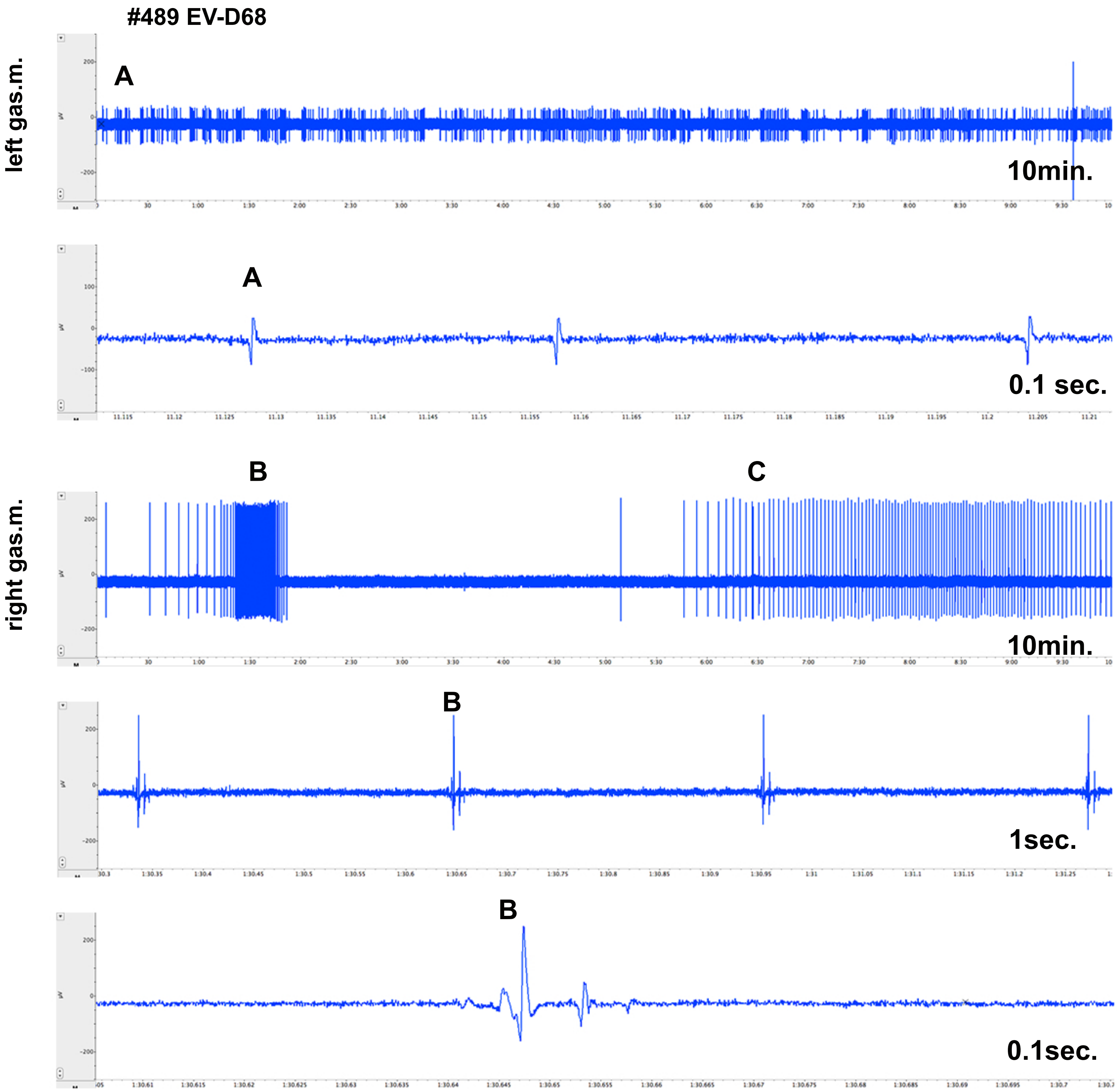
#489 EV-D68
left gas.m.
A
10min.
A
0.1 sec.
right gas.m.
B
C
10min.
B
1sec.
B
0.1sec.

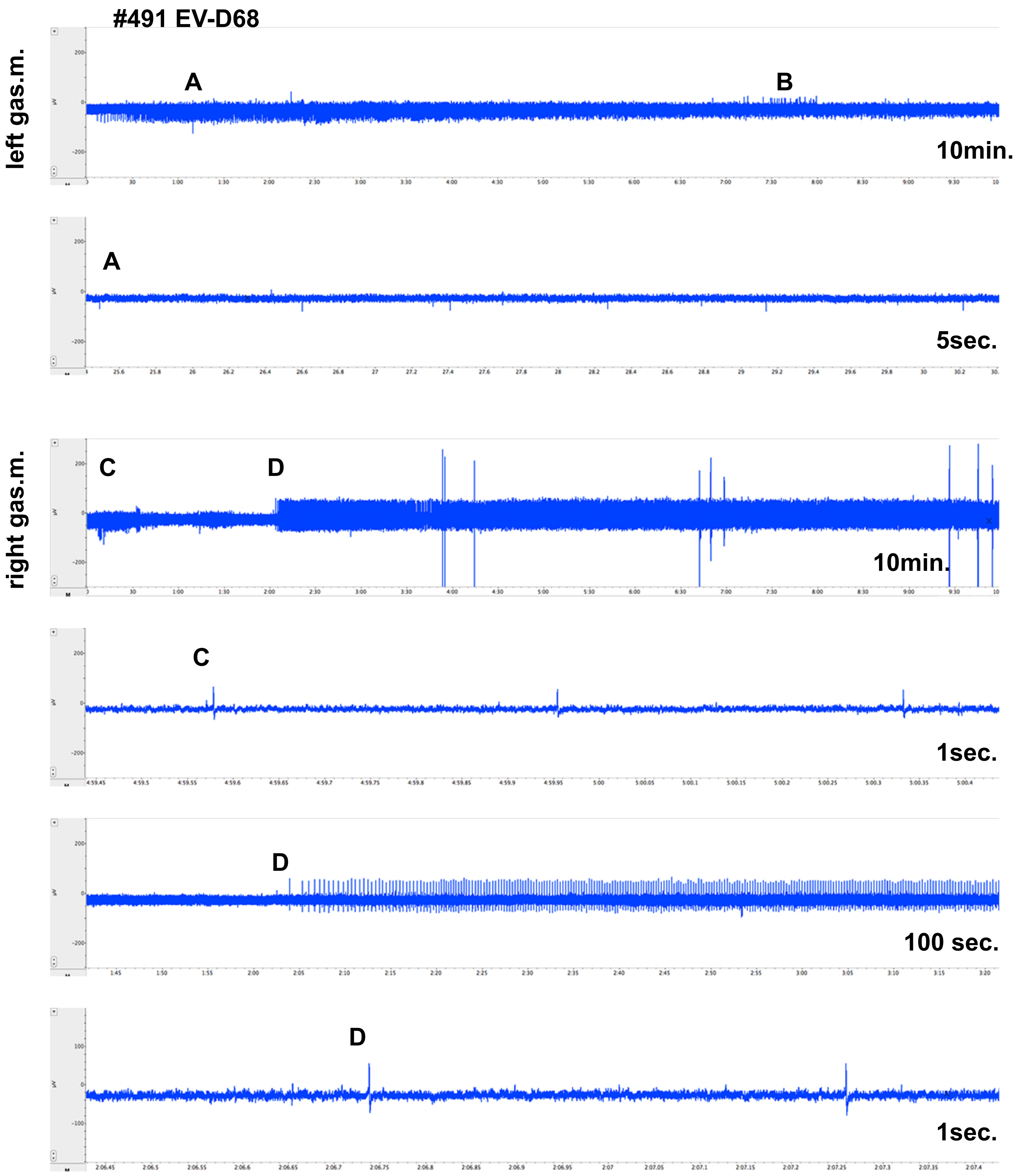
#491 EV-D68
left gas.m.
A
B
10min.
A
5sec.
right gas.m.
C
D
10min.
C
1sec.
D
100 sec.
D
1sec.

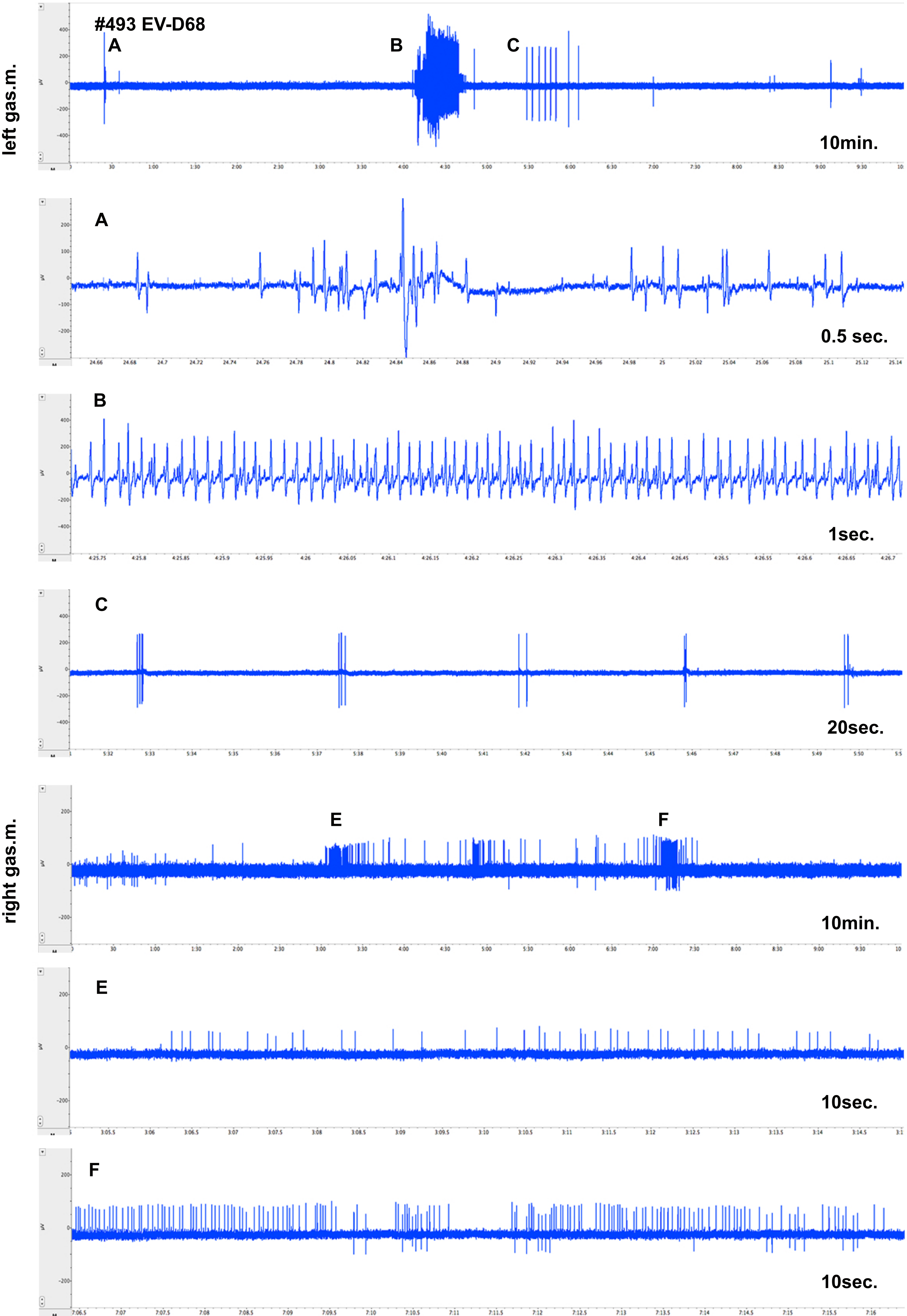

left gas.m.
#493 EV-D68
A
B
C
10min.
A
0.5 sec.
B
1sec.
C
20sec.
right gas.m.
E
F
10min.
E
10sec.
F
10sec.

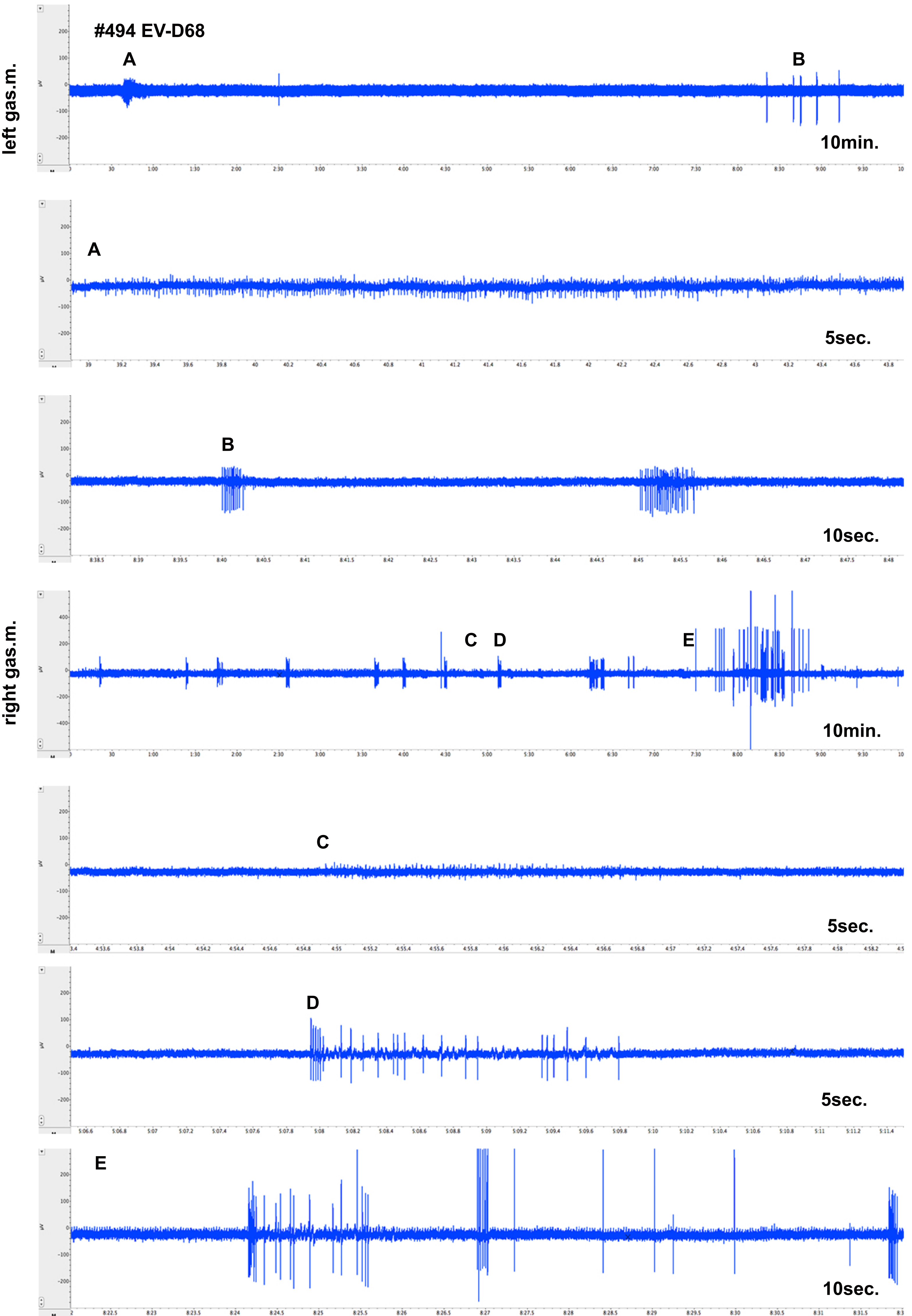
#494 EV-D68
left gas.m.
right gas.m.
A
B
C
D
E
10min.
5sec.
10sec.
10min.
5sec.
5sec.
10sec.
